# Tuberculosis treatment loss to follow-up in children exposed at home: A prospective cohort study

**DOI:** 10.7189/jogh.14.04194

**Published:** 2024-08-16

**Authors:** Meredith B Brooks, Brittney J van de Water, Leonid Lecca, Chuan-Chin Huang, Letizia Trevisi, Carmen Contreras, Jerome T Galea, Roger Calderon, Rosa Yataco, Megan Murray, Mercedes C Becerra

**Affiliations:** 1 Department of Global Health, Boston University School of Public Health, Boston, Massachusetts, USA; 2 Department of Global Health and Social Medicine, Harvard Medical School, Boston, Massachusetts, USA; 3 Connell School of Nursing, Boston College, Chestnut Hill, Massachusetts, USA; 4 Partners In Health / Socios En Salud, Lima, Peru; 5 Brigham and Women’s Hospital, Boston, Massachusetts, USA; 6 School of Social Work, University of South Florida, Tampa, Florida, USA

## Abstract

**Background:**

Loss to follow-up (LTFU) from tuberculosis (TB) treatment and care is a significant public health problem. It is important to understand what drives LTFU in children – a population whose treatment and management depend on an adult caregiver – to better provide support services to families affected by TB.

**Methods:**

We conducted a prospective cohort study of household contacts in Lima, Peru (2009–12). Using multilevel logistic regression analysis, we explored individual-level characteristics of children and their adult household members with TB disease to identify risk factors for LTFU among children initiated on treatment for TB.

**Results:**

A total of 154 child (0–14 years) household contacts were diagnosed with TB and initiated on treatment. While most (n = 133, 86.4%) had a successful outcome, 20 (13.0%) children were LTFU. Six (30.0%) children were LTFU within three months, nine (45.0%) between five to seven months, and three (15.0%) after seven months of treatment being initiated. In univariable analysis, children with index patients above 25 years of age had decreased odds of being LTFU (odds ratio = 0.26; 95% confidence interval = 0.08-0.84) compared to children with index patients 25 years or younger.

**Conclusions:**

In this cohort, more than 10% of children sick with TB who were exposed to the disease at home were LTFU. An integrated, family-centred TB prevention and management approach may reduce barriers to a child completing their course of TB treatment.

Tuberculosis (TB) remains a significant health challenge for children under 15 years of age, with over one million developing the disease annually [1–3] and 239 000 dying from it [4]. Proper diagnosis and treatment, however, can reduce mortality to less than 1% [5]. Ensuring treatment adherence remains essential in this sense, especially as loss to follow-up (LTFU) – defined as treatment being interrupted for two or more successive months [6] – leads to increased mortality [7], disease transmission, and drug resistance 8]. The frequency of child LTFU varies globally, ranging from 2.4% [9] to 14.9% [10]. Some risk factors for LTFU in children include HIV infection [11,12], previous TB disease [11,12], being under five years old [13], and limited financial resources [14].

Children face unique barriers to TB treatment adherence and completion compared to adults due to structural, community, household, and individual factors [15]. Dependence on caregivers for medication administration and clinic visits adds complexity to these challenges, particularly if caregivers lack knowledge or resources, or have competing priorities. Anticipated stigma and discrimination can also deter caregivers from seeking consistent treatment for their child [16]. Health system issues such as inadequate paediatric formulations, lack of child-friendly healthcare environments, large pill burdens, and long treatment durations contribute to poor adherence and LTFU [17,18]. Additionally, children’s developmental stages affect their understanding and management of long-term treatment, necessitating tailored interventions [15].

Children comprise 4% of notified cases in Peru, a country with a TB incidence of 151 per 100 000 population [3]. Peru’s Ministry of Health provides TB treatment and care for free through a network of health centres and hospitals. Yet despite reported treatment success rates of 86% nationally [3], there is a lack of information on treatment outcomes in children with TB, limiting the ability of the local TB programme to identify initiatives for improving care and providing support for this vulnerable population. With this in mind, we aimed to identify risk factors for LTFU within a cohort study of children treated for TB who lived with an adult with known TB in Lima, Peru. Understanding local factors driving LTFU will help tailor supportive services for children and their caregivers.

## METHODS

### Study design, setting, and population

From September 2009 to August 2012, a prospective household contact cohort study was conducted in Lima, Peru [19]. Patients aged 15 and older diagnosed with TB (‘index patients’) were recruited from 106 district health centres across Lima along with all of their household contacts. Index patients were diagnosed according to Peru’s national TB programme guidelines, which require either at least one of two positive sputum smears or a chest radiograph indicative of TB in the absence of a positive smear result. Study details, including the selection of participants from health centres, have previously been reported elsewhere [19].

Household contacts were assessed for TB at baseline. If they reported any symptoms of TB, including cough, night sweats, weight loss, or fever, they were referred to their local health centre for chest radiography and clinical evaluation for TB. Diagnosis for contacts under 18 years of age followed consensus guidelines for classifying TB in children [20]. Contacts diagnosed with TB within two weeks of the index patient were defined as co-prevalent cases; diagnoses beyond two weeks were defined as secondary cases. All diagnosed contacts received standard TB treatment per national guidelines. All contacts were re-visited at 2, 6, and 12 months.

### Predictor and outcome variables

Baseline characteristics for index patients and household contacts were age, gender, height (centimetres), weight (kilograms), underweight status (gender-specific body mass index-for-age Z-score ≤2, calculated per World Health Organization (WHO) Tables [21] for children), previous TB, student status, number of Bacille Calmette-Guerin (BCG) vaccine scars, alcohol consumption (non-drinker, light drinker (>0 g and <40 g or >0 and <3 alcoholic drinks per day), or heavy drinker (≥40 g or ≥3 alcoholic drinks per day)), smoking status (non-smoker, light smoker (1 cigarette per day), or heavy smoker (>1 cigarette per day)), presence of comorbidities (asthma, kidney disease, HIV, cardiac disease, diabetes, high blood pressure), diagnostic and drug sensitivity test (DST) results, and treatment regimen.

Additional characteristics were collected for index patients, such as education level (greater than high school or not), baseline smear status, and the presence of cavities on chest radiographs. The following characteristics were also collected for household contacts: tuberculin skin test (TST) result at baseline (national guidelines defined a positive TST by an induration with a diameter of ≥10 mm), baseline use of isoniazid preventive therapy, relationship to the index patient (child, sibling, or other), household socioeconomic status (classified as lowest, middle, and highest tertile from scores derived using principal components analysis of housing asset, weighted by household size [22]), number of individuals living in their home, and presence of any extra-pulmonary involvement. Standard WHO definitions for treatment outcomes [6] were used for individuals ≥15 years old, including cured, treatment completion, died, treatment failure, loss to follow-up, and not evaluable at the time. Treatment outcomes for children were recorded as indicated on the clinical chart by the treating clinician. Outcomes for children were not re-classified due to a lack of bacteriologic confirmation precluding standard outcome definitions from being met.

### Analysis

We conduct a secondary analysis of the household contact cohort study. This analysis included child contacts under 15 years diagnosed with TB disease and on treatment. We compared the characteristics of LTFU and non-LTFU children using χ2 or Fisher’s exact test for categorical variables and the Kruskal-Wallis test for continuous variables. We described the characteristics of each child who was LTFU, including their age, sex, relation to the index patient, duration (in months) between the index patient and child initiating TB treatment, duration (in months) between the child initiating TB treatment and being LTFU, number of individuals living in the household, DST results, and the age, sex, DST results, and treatment outcome of the index patient.

We then performed a multilevel univariable logistic regression that accounted for the clustering of child contacts within households. We excluded children whose treatment outcome was not evaluable at the time. Due to minimal missing data, we conducted a complete case analysis, whereby we assessed the association of each characteristic of the child or the index patient with child being LTFU. We reported our findings using odds ratios (ORs), 95% confidence intervals (CIs), and *P*-values. We included all variables associated with LTFU in univariable analysis (i.e. with a *P*-value ≤0.05) in a multivariable model, along with other important characteristics, such as those previously linked to treatment outcomes and being LTFU (age, sex, prior history of TB, and household socioeconomic status). We checked all variables included in the multivariable model for collinearity by assessing variance inflation factors. We conducted all analyses using SAS, version 9.4 (SAS Institute, Cary, North Carolina, USA).

### Ethics approval and consent to participate

The Institutional Review Board of the Harvard School of Public Health (Boston, MA, USA) and the Research Ethics Committee of the National Institute of Health of Peru (Lima, Peru) approved the initial household contact cohort study. Study participants or their guardians provided voluntary, written informed consent prior to study enrollment. All procedures were performed in accordance with relevant local guidelines and regulations. The Boston University Institutional Review Board (H-43041) deemed our secondary analysis of this de-identified data as non-human subjects research; therefore, we did not require separate ethical approval.

## RESULTS

During the study period, 154 child contacts aged 0–14 were diagnosed with TB and initiated treatment. Seventy-two (46.8%) children were 0–4 years, 38 (24.7%) were 5–9 years, and 44 (28.6%) were 10–14 years old (**Table 1**). Eighty-one (52.6%) children were female, 75 (48.7%) had a positive TST at baseline, and 30 (19.5%) reported having received a course of isoniazid chemoprophylaxis at baseline. Over half (n = 90, 58.4%) of the children’s index TB patient in the household was a parent. Thirty-nine (25.3%) children were diagnosed with TB disease within two weeks of the index TB patient, while 115 (74.7%) were diagnosed during the year of follow-up. Only 34 (22.1%) children had microbiological confirmation of TB disease, 7 (4.6%) had drug-resistant TB, and 23 (15.0%) had extrapulmonary involvement. Most children were successfully treated (n = 133, 86.4%) (**Table 2**). While no child died or had treatment failure, 20 (13.0%) were LTFU and one (0.7%) was unable to be evaluated.

**Table 1:** Baseline demographics and clinical characteristics of 154 children treated for TB*

Variables	All children (n = 154)†	Children who were LTFU (n = 20)†	Children with other treatment outcomes (n = 133)†	*P*-value
Age in years, MD (IQR)	5 (3–10)	4.5 (2–10)	5.5 (3–10)	0.51^‡^
Age in years				0.93
*0–4*	72 (46.8)	10 (50.0)	62 (46.3)	
*5–9*	38 (24.7)	5 (25.0)	33 (24.6)	
*10–14*	44 (28.6)	5 (25.0)	39 (29.1)	
Female sex	81 (52.6)	13 (65.0)	68 (50.8)	0.23
Underweight (n = 153)	4 (2.6)	0 (0)	4 (3.0)	1.00§
Previous TB	6 (3.9)	1 (5.0)	5 (3.7)	0.57§
Current student	90 (58.4)	9 (45.0)	81 (60.5)	0.19
TST positive at baseline	75 (48.7)	11 (55.0)	64 (47.8)	0.55
IPT use	30 (19.5)	4 (20.0)	26 (19.4)	0.95
BCG scars				0.83
*0*	34 (22.1)	4 (20.0)	30 (22.4)	
*1*	118 (76.6)	16 (80.0)	102 (76.1)	
*2*	2 (1.3)	0 (0.’)	2 (1.5)	
Socioeconomic status				0.57
*Lower tertile*	59 (40.7)¶	8 (53.3)║	51 (39.2)**	
*Middle and higher tertile*	86 (59.3)¶	7 (46.7)║	79 (60.8)**	
Relation to index patient				0.84
*Child*	90 (58.4)	12 (60.0)	78 (58.2)	
*Sibling*	26 (16.9)	4 (20.0)	22 (16.4)	
*Other*	38 (24.7)	4 (20.0)	34 (25.4)	
Asthma	10 (6.5)	1 (5.0)	9 (6.7)	0.77
Number individuals living in home, MD (IQR)	6 (4–8)	5.5 (3.5–7.5)	6 (4–8)	0.44^‡^
Timing of TB				0.26
*Co-prevalent case*	39 (25.3)	3 (15.0)	36 (26.9)	
*Secondary case*	115 (74.7)	17 (85.0)	98 (73.1)	
Microbiological confirmation	34 (22.1)	2 (10.0)	32 (23.9)	0.16
Drug-resistant TB	7 (4.6)	1 (5.0)	6 (4.5)	1.00§
Extrapulmonary involvement	23 (15.0)	5 (25.0)	18 (13.4)	0.18

BCG – Bacille Calmette-Guerin vaccine, IPT – isoniazid preventive treatment, IQR – interquartile range, LTFU – loss to follow-up, MD – median, TB – tuberculosis, TST – tuberculin skin test

*Presented as n (%) unless specified otherwise.

†Child characteristics: additionally, fewer than five children had cardiac disease or high blood pressure; no child had HIV co-infection, diabetes, or kidney disease.

^‡^Kruskal-Wallis test.

§Fisher exact test.

¶n = 145.

║n = 15.

**n = 130.

**Table 2:** Treatment outcomes of 154 children treated for TB

Treatment outcomes for child contacts	n (%)
Successful outcomes	133 (86.4)
*Cured*	91 (59.1)
*Treatment completion*	42 (27.3)
Died	0 (0)
Treatment failure	0 (0)
Loss to follow-up	20 (13.0)
Not evaluable at time	1 (0.7)

Compared to non-LTFU children, LTFU children were mostly younger, female, from lower socioeconomic households, and secondary cases, and they had less microbiological confirmation and more extrapulmonary involvement (**Table 1**).

The 154 children lived in 127 households; the number of children diagnosed with TB per household ranged from one to seven, with 111 (87.4%) households having only one child with TB, eleven (8.7%) households having two, three (2.4%) households having three, and one (0.8%) household each having five and seven children diagnosed with TB. Most index TB patients were female (n = 73, 57.5%) and younger (median (MD) = 28 years, IQR = 20–37) (**Table 3**). Of the 127 index patients, 33 (26.0%) previously had TB disease, 95 (74.8%) had a positive baseline smear status, 45 (35.4%) had a cavity present on chest x-ray findings, and 112 (88.9%) were initiated on a drug-susceptible TB treatment regimen. Index patients with at least one child contact who was LTFU were significantly younger (MD = 21 years, IQR = 19–31 vs MD = 29 years, IQR = 21–38; *P* = 0.03) and had fewer BCG scars (n = 17, 100% vs n = 86, 78.2% with zero or one scar; *P* = 0.03) than index patients with non-LTFU child contacts.

**Table 3:** Baseline demographics and clinical characteristics of 127 index TB patients of the child contacts treated for TB*

Variables	All index patients with at least one child contact with TB (n = 127)†	Index patient with at least one child contact who was LTFU (n = 17)†	Index patient with a child contact with other treatment outcomes (n = 110)†	*P*-value
Age in years, MD (IQR)	28 (20–37)	21 (19–31)	29 (21–38)	0.03‡
Age in years				0.23
*16–19*	25 (19.7)	7 (41.2)	18 (16.4)	
*20–29*	47 (37.0)	5 (29.4)	42 (38.2)	
*30–39*	31 (24.4)	4 (23.5)	27 (24.6)	
*40–49*	16 (12.6)	1 (5.9)	15 (13.6)	
*50–59*	3 (2.4)	0 (0.0)	3 (2.7)	
*≥60*	5 (3.9)	0 (0.0)	5 (4.6)	
Female sex	73 (57.5)	11 (64.7)	62 (56.4)	0.52
Underweight (n = 127)	3 (2.4)	0 (0)	3 (2.8)	1.00§
Previous TB	33 (26.0)	5 (29.4)	28 (25.5)	0.73
Current student (n = 126)	13 (10.3)	2 (11.8)	11 (10.1)	0.83
Education > high school (n = 126)	23 (18.3)	3 (17.7)	20 (18.4)	0.95
Number of BCG scars				0.03
*0*	18 (14.2)	1 (5.9)	17 (15.5)	
*1*	85 (66.9)	16 (94.1)	69 (62.7)	
*2 or more*	24 (18.9)	0 (0.0)	24 (21.8)	
Smoking status				1.00§
*Non-smoker*	124 (97.6)	17 (100.0)	107 (97.3)	
*Smoker*	3 (2.4)	0 (0.0)	3 (2.7)	
Drinking status (n = 123)				0.08
*Non-drinker*	76 (61.8)	9 (52.9)	67 (63.2)	
*Light-drinker*	34 (27.6)	8 (47.1)	26 (24.5)	
*Heavy-drinker*	13 (10.6)	0 (0.0)	13 (12.3)	
Baseline smear status				0.42
*Negative*	32 (25.2)	7 (41.2)	25 (22.7)	
*+*	35 (27.6)	3 (17.7)	32 (29.1)	
*++*	25 (19.7)	3 (17.7)	22 (20.0)	
*+++*	35 (27.6)	4 (23.5)	31 (28.2)	
Asthma (n = 126)	12 (9.5)	3 (17.7)	9 (8.3)	0.22
Kidney disease (n = 126)	5 (4.0)	1 (6.3)	4 (3.6)	0.62§
Cavity on chest radiograph	45 (35.4)	7 (41.2)	38 (34.6)	0.60
Microbiological confirmation	122 (96.1)	16 (94.1)	106 (96.4)	0.52§
Regimen type (n = 126)				0.93
*Drug-susceptible*	112 (88.9)	15 (88.2)	97 (89.0)	
*Drug-resistant*	14 (11.1)	2 (11.8)	12 (11.0)	

BCG – Bacille Calmette-Guerin vaccine, IQR – interquartile range, LTFU – loss to follow-up, MD – median, TB – tuberculosis

*Presented as n (%) unless specified otherwise

†Index patient characteristics: fewer than five patients had HIV co-infection, cardiac disease, diabetes, or high blood pressure.

‡Kruskal-Wallis test.

§Fisher exact test.

Among the 20 children who were LTFU, 12 (60.0%) were five years or younger, while most index patients were under 30 years of age (n = 15, 75.0%) and female (n = 14, 70.0%) (**Table 4**). The parents of more than half of the LTFU were index patients (n = 12, 60.0%); among them, ten (83.3%) were the child’s mother, while nine (75.0%) children were five years or younger. Of those ten mothers, seven (70.0%) were 25 years of age or younger. Only two of the index patients were also LTFU; both were siblings of the child contact and were only 18 years old. Most index patients of children who were LTFU were successfully treated (n = 16, 80.0%), while most children developed TB disease within three months after the index patient was diagnosed (n = 15, 75.0%). Only six (30.0%) children were LTFU within three months of treatment initiation. Another nine (45.0%) children were LTFU between five and seven months and three (15.0%) were declared LTFU over seven months after treatment initiation.

**Table 4:** Characteristics of the 20 children treated for TB who were lost to follow-up

	Child characteristics								Index patient characteristics					
**Number**	**Age**	**Sex**	**Relation to index**	**Number of people living in household**	**DS or DR**	**Extrapulmonary involvement**	**Duration from index diagnosis to child treatment initiation in months**	**Duration from child’s treatment initiation until LTFU in months**	**Age**	**Sex**	**Drinker**	**DS or DR**	Treatment outcome*
1	0	F	Child	8	DS	No	<1	<1	31	F	No	DS	Successful
2	0	F	Child	3	DS	No	2	7	19	F	Yes	DS	Successful
3	1	M	Child	6	DS	No	<1	6	19	F	No	DS	Successful
4	1	F	Child	6	DS	No	1	>7	21	F	No	DS	Successful
5	2	F	Child	3	DS	No	3	2	25	F	No	DS	Successful
6	2	F	Sibling	7	DS	No	3	>7	18	F	Yes	DR	LTFU
7	3	F	Other	5	DS	Yes	3	4	17	F	No	DS	Successful
8	3	F	Sibling	4	DS	No	2	1	19	M	No	DS	Active treatment
9	4	F	Child	3	DS	Yes	9	5	27	M	Yes	DS	Successful
10†	4	F	Child	17	DS	No	<1	6	23	F	Yes	DS	Successful
11	5	F	Child	5	DS	Yes	1	>7	20	F	Yes	DR	Successful
12†	5	M	Child	17	DS	No	<1	6	23	F	Yes	DS	Successful
13†	6	F	Other	17	DS	No	1	3	23	F	Yes	DS	Successful
14	7	M	Child	3	DS	Yes	<1	5	33	F	Yes	DS	Active treatment
15	9	M	Sibling	3	DS	Yes	12	2	16	M	No	DS	Successful
16	11	M	Sibling	4	DS	N	<1	7	18	M	No	DS	LTFU
17†	12	M	Other	17	DS	No	1	7	23	F	Yes	DS	Successful
18	13	F	Child	4	DS	No	12	6	35	M	Yes	DS	Successful
19	14	M	Child	6	DR	No	4	4	38	F	N	DS	Successful
20	14	F	Other	7	DS	No	7	1	46	M	Yes	DS	Successful

DR – drug-resistant tuberculosis, DS – drug-susceptible tuberculosis, F – female, LTFU – loss to follow-up, M – male

*Successful outcome: defined as cure or treatment completion per WHO guidelines. Active treatment: on last course of treatment and defined as still on treatment by the doctor.

†Four children were from the same household and connected to the same index patient.

One child whose treatment outcome was not evaluated at the time data collection was closed was excluded from the final analysis, leaving 153 children in our sample. In univariate analysis, the age of the index patient was associated with the child being LTFU; children of index patients greater than 25 years of age had decreased odds of being LTFU compared to children of index patients 25 years or younger (OR = 0.26; 95% CI = 0.08–0.84) (**Table 5**). When controlling for the age and sex of the child and the household socioeconomic status, the odds of a child of an index patient greater than 25 years of age being LTFU compared to a child of an index patient 25 years or younger shifted to an OR of 0.35 (95% CI = 0.10–1.20). We did not include the history of TB in the multivariable model due to having too few observations. Assessment of variance inflation factors indicated no significant collinearity among included variables.

**Table 5:** Univariable analysis of risk factors for loss-to follow up among child household contacts treated for drug-susceptible TB (n = 153)

	Univariable analysis		Multivariable analysis	
**Characteristics**	**OR (95% CI)**	***P*-value**	**Adjusted OR (95% CI)**	***P*-value**
**Characteristics of the children treated for drug-susceptible tuberculosis**				
Age in years*	0.96 (0.85–1.09)	0.55	0.97 (0.85–1.12)	0.68
Age in years				
*0–4*	ref	ref		
*5–9*	0.78 (0.20–3.06)	0.72		
*10–14*	0.74 (0.20–2.76)	0.64		
Female sex	1.72 (0.57–5.20)	0.32	1.93 (0.55–6.81)	0.29
Previous TB	1.54 (0.13–18.85)	0.73		
Current student	0.47 (0.16–1.42)	0.17		
TST positive at baseline	1.32 (0.45–3.88)	0.61		
IPT use	1.12 (0.28–4.40)	0.87		
Number of BCG scars ≥1	1.02 (0.27–3.86)	0.97		
Socioeconomic status				
*Middle and higher tertile*	ref	ref	ref	ref
*Lower tertile*	1.73 (0.54–5.60)	0.34	1.82 (0.52–6.42)	0.34
Relation to index patient				
*Child*	ref	ref		
*Sibling*	1.05 (0.25–4.40)	0.94		
*Other*	0.70 (0.17–2.78)	0.60		
Asthma	0.81 (0.08–8.63)	0.85		
Number individuals living in home*	0.97 (0.83–1.13)	0.65		
Timing of TB				
*Co-prevalent case*	ref	ref		
*Secondary case*	1.86 (0.44–7.90)	0.39		
Microbiological confirmation	0.36 (0.07–1.95)	0.23		
Regimen type				
*Drug-susceptible*	ref	ref		
*Drug-resistant*	1.08 (0.09–13.73)	0.95		
Extrapulmonary involvement	2.25 (0.60–8.46)	0.22		
**Characteristics of the index TB patients**				
Age in years*	0.93 (0.87–1.00)	0.05		
Age in years				
16–25	ref	ref	ref	ref
>25	0.26 (0.08–0.84)	0.03	0.35 (0.10–1.20)	0.09
Female sex	1.37 (0.42–4.42)	0.59		
Previous TB	1.06 (0.30–3.74)	0.93		
Current student	1.30 (0.21–7.99)	0.77		
Education > high school	0.97 (0.22–4.33)	0.96		
Number of BCG scars ≥1	3.64 (0.38–35.06)	0.25		
Drinking status				
*Non-drinker*	ref	ref		
*Drinker*	1.69 (0.54-5.23)	0.35		
Baseline smear status				
*Negative*	ref	ref		
*+*	0.31 (0.06–1.58)	0.15		
*++*	0.47 (0.09–2.48)	0.36		
*+++*	0.62 (0.15–2.57)	0.49		
Asthma	1.90 (0.37–9.72)	0.43		
Kidney disease	1.91 (0.14–26.39)	0.62		
Cavity on chest radiograph	1.31 (0.42–4.04)	0.63		
Microbiological confirmation	0.41 (0.03–6.34)	0.51		
Regimen type				
*Drug-susceptible*	ref	ref		
*Drug-resistant*	1.18 (0.19–7.13)	0.85		

BCG – Bacille Calmette-Guerin vaccine, CI – confidence interval, IPT – isoniazid preventive treatment, OR – odds ratio, ref – reference, TB – tuberculosis, TST – tuberculin skin test

*Variables are assessed as one-unit offsets from the mean. Malnutrition and smoking status are not included because model did not converge due to small event number.

## DISCUSSION

The age of the index TB patient in the household was associated with a child contact being LTFU; specifically, the younger the index patient (down to age 15), the higher the odds of the child contact being LTFU. Most children who were LTFU were five years or younger and had young, female caregivers (often the mother) who, as the index patient, had a successful treatment outcome. Young mothers have a myriad of responsibilities, including taking their own treatment and attending health visits, earning wages, or having other children to care for; they may also be less financially stable, which may have led to difficulty in attending clinic visits or ensuring their child completes treatment [23–25]. While this finding is not well documented within the TB literature, research on HIV suggests a strong association between the young age of a mother/caregiver and increased LTFU of children/infants during HIV treatment [23,26,27].

We also found that, in one-third of children, LTFU occurred within three months of treatment initiation, which is consistent with other reports of children LTFU [28]. Additionally, over 40% of children were LTFU between five and seven months post-treatment initiation. Standard treatment for drug-susceptible TB is six months, but this may be slightly extended if there are gaps in adherence. Being marked LTFU during the five- to seven-month time frame may indicate that a child’s caregiver picked up their last month of medications, but did not return to the health facility after the child completed treatment to have a formal treatment outcome recorded in their medical chart. Similar types of recording errors have been documented elsewhere [29,30]. Additionally, three children were declared to be LTFU over seven months after treatment initiation, which may indicate a systems-level issue related to the timely charting of patient records, where patients completed their treatment earlier and the final visit was not appropriately logged, or where patients were available via phone contact but did not come into the clinic for a visit, due to which they were not noted to be LTFU until this time frame [29,31,32]. Shorter regimens are beginning to be recommended for children, including four-month regimens for non-severe drug-sensitive TB [33]. These will likely improve adherence and help drive down LTFU in children, especially among individuals who are LTFU in the later stages of treatment. Although the WHO has recently provided updated guidelines for TB diagnostics, treatment, and prevention, Peru’s national protocols have lagged, making these findings pertinent to include in forthcoming national guidelines for the management of TB in children [34,35].

Eighty per cent of children with TB were diagnosed within three months of the index TB patient being diagnosed. This is in line with other existing literature [36,37], indicating that rapid and perhaps even frequent evaluations of household contacts are important to promptly identify individuals who progress to TB disease [38].

Overall, most children had a successful outcome, but almost 13% were LTFU. This is consistent with other studies reporting treatment outcomes for children with DS-TB globally [9,10]. In this cohort, children did not experience any other poor treatment outcomes such as death or treatment failure, indicating that increasing efforts to reduce LTFU in this vulnerable population will lead to higher rates of treatment success.

Due to children’s reliance on adult caregivers for transportation to appointments, medication dispensing, treatment support and adherence, and overall dependence regardless of illness, taking a family-centred care (FCC) approach for TB care and management would be beneficial. This approach builds upon the WHO’s End TB Strategy Pillar One, emphasizing the provision of integrated, patient-centred care and prevention [39]. Poor treatment supervision and household financial burdens have been identified as barriers to TB treatment adherence and completion; therefore, providing FCC to families of children with TB and households with multiple individuals with TB may involve providing families with more robust and collective treatment support services or economic support in the form of necessary accommodations or transportation assistance to health appointments, as well as shifting the burden of responsibility for access to care and treatment from caregivers to health systems [40–43]. Clustering multiple visits of all TB patients in a household to the health centre or simultaneously providing treatment could be valuable for adherence and retention in care, potentially for parents who themselves are being treated for TB as well, as our findings indicated that even though children were LTFU, the adult index patient often still had a successful treatment outcome. Using an FCC approach in contact tracing [44,45] would ensure that families are properly screened throughout an index TB patient’s treatment to identify other household members with TB as early as possible. An FCC approach once treatment is initiated may facilitate better completion of treatment for all family members with TB, ultimately leading to better treatment outcomes, particularly among young children. Future research should focus on children with a parent, especially young mothers who are also sick with TB, and the identification of tailored, FCC interventions to address their needs.

The study has several limitations. Children were often not able to meet standard TB treatment outcome definitions due to a lack of microbiological confirmation or an inability to provide a sputum sample. Thus, we used the treatment outcome as recorded in the medical chart by the child’s treating clinician. While this may have resulted in outcome misclassification, its overall categorisation as successful or unsuccessful will likely remain the same. This was also unlikely to have affected children who were LTFU. Due to the study taking place from 2009–12, we could not reach out to study staff and clinicians to obtain insights into why several children were declared LTFU after seven months post-treatment initiation, despite treatment for drug-susceptible TB being only six months. Similarly, we do not know what additional household or social support local families received outside of their participation in the study. Finally, we only had a small sample of 20 children who were LTFU, making it difficult to identify strong associations and limiting the generalisability of our findings.

## CONCLUSIONS

We observed that LTFU is high among children treated for TB who live in a household with an adult with TB; that children had higher odds of being LTFU if the index patient in their home was 25 years or younger; and that LTFU occurred throughout all stages of TB treatment. Integrating a family-centred prevention and management approach into TB programmes could reduce barriers for children completing TB treatment at any stage of their treatment course, ultimately leading to less morbidity and mortality. However, future research is warranted to understand, integrate, and refine this model of care into existing TB programmes and to identify opportunities to support young caregivers with TB.
